# Metastatic malignant melanoma with neuroendocrine differentiation: a case report and review of the literature

**DOI:** 10.1186/s13256-020-02367-z

**Published:** 2020-03-31

**Authors:** Carl Christofer Juhlin, Jan Zedenius, Felix Haglund

**Affiliations:** 1 Department of Oncology-Pathology, Karolinska Institutet, Stockholm, Sweden; 2 Department of Pathology and Cytology, Karolinska University Hospital, Stockholm, Sweden; 3 Department of Breast, Endocrine Tumors and Sarcoma, Karolinska University Hospital, Stockholm, Sweden; 4 Department of Molecular Medicine and Surgery, Karolinska Institutet, Stockholm, Sweden

**Keywords:** Malignant melanoma, Neuroendocrine differentiation, Synaptophysin, Neuroendocrine cancer, Carcinoma of unknown primary, Metastasis

## Abstract

**Background:**

Metastatic neuroendocrine carcinoma often presents as carcinoma of unknown primary. Although most cases display immunohistochemical positivity for neuroendocrine markers, subsets of cases display reduced or negative expression for some of these proteins. The identification of metastatic neuroendocrine carcinomas is even more complicated by the occurrence of unrelated tumor types with focal neuroendocrine differentiation.

**Case presentation:**

Our patient was a 74-year-old man of Middle Eastern ethnicity. An initial biopsy of a soft tissue metastasis displayed a neuroendocrine profile indicative of a metastatic neuroendocrine carcinoma, positive for CD56 and synaptophysin, and focally for ISL LIM homeobox 1 and insulinoma-associated protein 1. The Ki-67 index was 50%. Chemotherapy was initiated, but our patient progressed. Scrapings from a pathological hip fracture 3 months later revealed focal synaptophysin immunoreactivity and widespread melanoma antigen, human melanoma black 45, and SOX10 positivity, which are indicative of metastatic malignant melanoma with focal neuroendocrine differentiation.

**Conclusions:**

Malignant melanoma may display neuroendocrine differentiation, and the entity should be considered a rare differential diagnosis when assessing biopsies of suspected neuroendocrine carcinomas.

## Background

The distinction between a neuroendocrine tumor (NET) and various epithelial and non-epithelial tumors with focal neuroendocrine differentiation is an established clinical dilemma. The diagnosis relies on the identification of clear-cut immunoreactivity towards neuroendocrine markers such as chromogranin A (CgA), synaptophysin (SYP), and CD56, but the expression of one or several of these markers might also be found in subsets of non-NETs [1–6]. As these tumors are biologically distinct from NETs, the associated treatment options vary; therefore, proper recognition is imperative for the patient’s overall prognosis. The distinction between a metastatic NET and non-NET tumor with focal neuroendocrine differentiation could be particularly difficult when assessing biopsy material of metastatic deposits from a cancer of unknown primary (CUP), given the general privation of tissue material for immunohistochemical purposes.

CgA and SYP are proteins associated with vesicles of neurons and endocrine cells, with established roles for the secretion of various peptides mediated through exocytosis [7, 8]. NETs are usually positive for both markers, reflecting the underlying secretory potential of these tumor types. CD56, also known as neural cell adhesion molecule (NCAM), is important for inducing neurite outgrowth in neurons, but is also generally present in NETs [9]. Not surprisingly, non-NET carcinomas might also express CgA, SYP, and/or CD56 to a variable extent. This phenomenon is well described for a variety of tumor types – and sometimes also coupled to specific clinical characteristics and patient prognosis. For example, prostatic adenocarcinoma with focal neuroendocrine differentiation is associated with worse clinical outcome, whereas the same correlation is not found in adenocarcinomas of the breast [2, 3].

In this case report, we describe a patient with disseminated metastatic disease and depict how the manifestation of a malignant melanoma with neuroendocrine features mimicked a neuroendocrine carcinoma (NEC) from histological and immunophenotypic aspects, including the clinical consequences.

## Case presentation

Our patient was a 74-year-old man of Middle Eastern ethnicity with a previous medical history including hypertension, benign prostatic hyperplasia, polycythemia vera, and duodenal ulcers. He received metoprolol (100 mg daily) as well as aspirin (75 mg daily), had a history of tobacco smoking, but ceased tobacco use 15 years prior to the current admission. He did not consume alcohol. He was born in Iraq and arrived in Sweden 2002. He was previously employed as a medical secretary but he had retired at the time of admission. His previous social history is largely unknown, but he was married, and he had three children (two sons and one daughter) according to previous medical files.

In the early spring of 2019, he developed dyspnea and flank pain, and following investigations at a secondary care institution, a subsequent radiological computed tomography (CT) examination revealed at least six lesions in his liver, of which the largest deposit measured 13 mm. In addition, a 50 mm large soft tissue expansion in conjunction to his chest wall with engagement and destruction of the eighth rib was observed. In his lungs, 13 nodular lesions were detected of which the largest measured 8 mm. A bone metastasis to the right acetabular region was also visualized, in addition to a 10 mm large lesion in his left adrenal gland. The latter nodule was present already on a radiological examination performed 12 years earlier and found stationary with no increase in overall size; it was therefore believed to be benign. To summarize, the lesions observed were highly suspicious for metastatic deposits from a CUP. An ultrasonography-guided core-needle biopsy of the chest wall lesion was performed, and a diagnosis of metastatic NEC was rendered. The material was immediately sent for second opinion consultation at our tertiary unit.

**Fig. 1:**
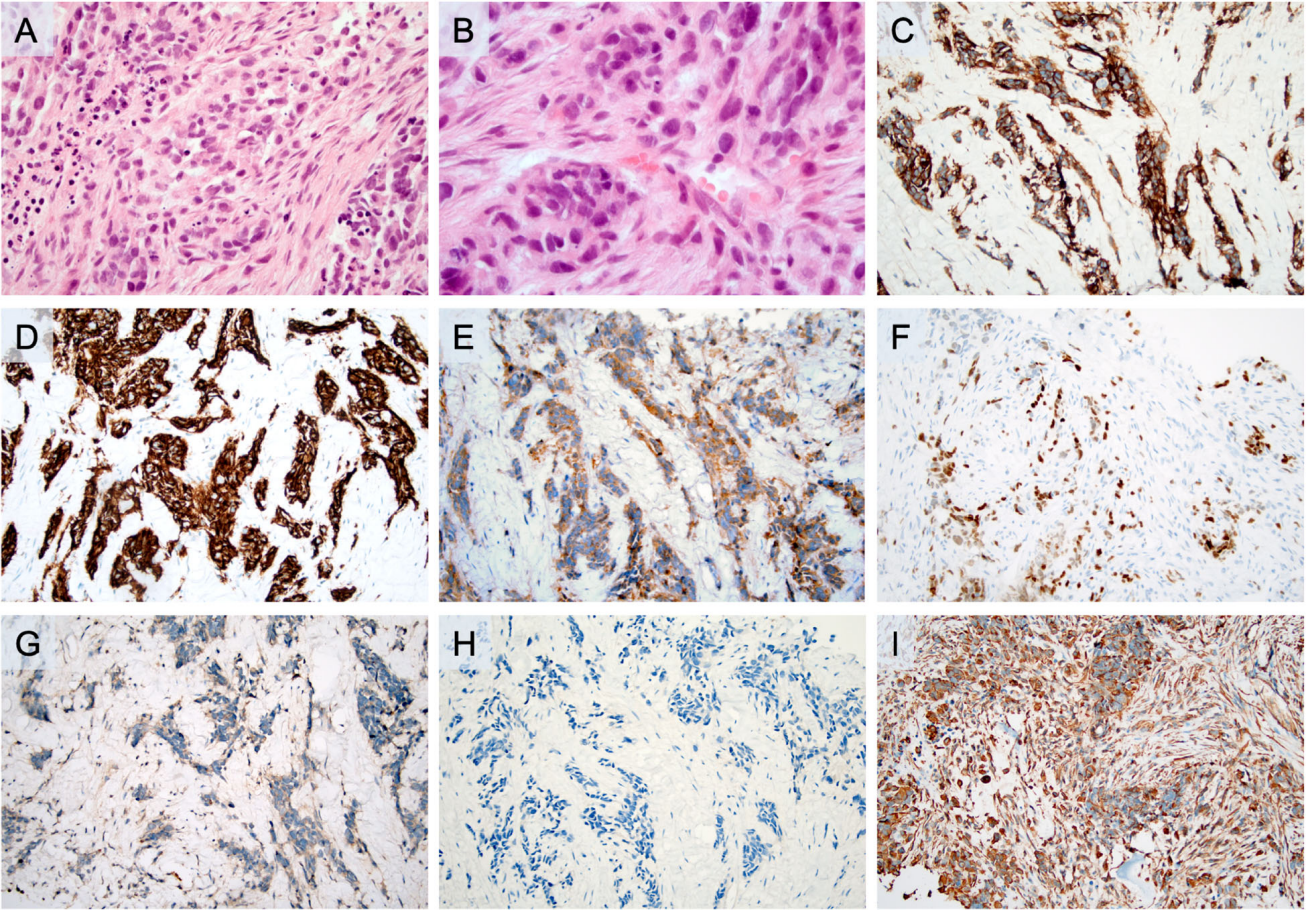
Microscopic findings of the initial core-needle biopsy from the 50 mm soft tissue expansion adjacent to the chest wall. This lesion was initially believed to constitute a metastatic neuroendocrine carcinoma. All microscopic images are magnified × 200 unless otherwise stated. **a**. Routine hematoxylin and eosin staining depicting a tumor with a predominant solid growth pattern infiltrating the surrounding stroma. **b**. Routine hematoxylin and eosin section at × 600 magnification, illustrating nuclear pleomorphism. **c**. Widespread cytoplasmic synaptophysin immunoreactivity. **d**. Uniform CD56 immunoreactivity. **e**. Diffuse cytoplasmic insulinoma-associated protein 1 staining. **f**. Focal ISL LIM homeobox 1 nuclear staining (subsets of tumor cells). **g**. Negative staining for melanoma antigen. **h**. Negative staining for human melanoma black 45. **i**. Diffuse positivity for vimentin

The histopathological examination revealed a tumor with a predominant solid growth pattern, diffusely infiltrating the surrounding fibrotic stroma (Fig. 1a). The tumor cells displayed round to slightly polygonal nuclei, focally pleomorphic, with an evenly distributed chromatin (Fig. 1b). Several nuclear inclusions were observed, as were comedo-like tumor necrosis and apoptotic bodies. No prominent nucleoli or cytoplasmic pigmentation was noted. The immunohistochemical analysis was carried out in a routine pathology laboratory setting with clinically accredited antibodies and standardized methodology. The profiling was consistent with a tumor uniformly positive for SYP and CD56 (Table 1; Fig. 1c, d). Unexpected and diffuse cytoplasmic immunoreactivity was noted for insulinoma-associated protein 1 (INSM1), and subsets of cells were also positive for ISL LIM homeobox 1 (ISLET1; Fig. 1e, f). The tumor was negative for melanoma antigen (Melan A) and human melanoma black 45 (HMB45; Fig. 1g, h) but was strongly positive for vimentin (Fig. 1i). Scattered tumor cells were positive for cytokeratin OSCAR. The tumor cells were negative for: pan-cytokeratin (CK MNF 116); cytokeratins 5, 7, 19, and 20; epithelial membrane antigen (EMA); GATA binding protein 3 (GATA3); hepatocyte-specific antigen; P63; prostate-specific antigen (PSA); thyroid transcription factor 1 (TTF1); paired box 8 (PAX8); caudal type homeobox 2 (CDX2); carcinoembryonic antigen (CEA); CgA; secretagogin; steroidogenic factor 1 (SF1); inhibin alpha; glucagon-like peptide 1 (GLP1); and pancreatic and duodenal homeobox 1 (PDX1) (data not shown). The Ki-67 index was 50%. The P53 immunoreactivity was focal and weak, not suspicious for an underlying *TP53* gene alteration. The pathology report concluded the presence of a metastatic, high-grade malignant tumor with a neuroendocrine profile and unknown primary location; the absent CgA immunoreactivity could argue in favor of a NEC because this tumor entity occasionally downregulates CgA expression upon tumoral de-differentiation. The keratin-negative profile did, however, also raise the suspicion of a non-NET with a neuroendocrine differentiation, but the paraffin block was exhausted after cutting material for immunohistochemistry; therefore, no additional analyses could be performed.

**Table 1:** Immunohistochemical details of the metastatic lesions

			*Immunohistochemical markers*										
Time point	Tissue investigated	Procedure	CgA	SYP	CD56	INSM1	ISLET1	Cyto-keratins	Vimentin	Melan A	HMB45	SOX10	Diagnosis
April 2019	Chest wall	Core-needle biopsy	–	+ (100%)	+ (100%)	+ (100%)	+ (10%)	– *	+ (100%)	–	–	n.d.	**NEC**
June 2019	Femur	Surgical excision	–	+ (15%)	+ (100%)	n.d.	n.d.	–	+ (100%)	+ (30%)	+ (30%)	+ (100%)	**MM-NE**

*CgA* chromogranin A, *HMB45* human melanoma black 45, *INSM1* insulinoma-associated protein 1, *ISLET1* ISL LIM homeobox 1, *Melan A* melanoma antigen, *MM-NE* malignant melanoma with neuroendocrine differentiation, *n.d.* not determined, *– *negative staining, *NEC* neuroendocrine carcinoma, *SYP* synaptophysin

*Focal cytokeratin OSCAR immunoreactivity noted in few tumor cells – uncertain significance

Our patient was discussed at a multidisciplinary conference and was recommended to commence chemotherapy (carboplatin and etoposide). On admission to our hospital, he was confined to a wheelchair and in considerable pain from his groin and lumbar regions. His groin was investigated, and a 20 mm enlarged lymph node was palpable and was assumed to be part of his disseminated disease. He had substantial bilateral pitting edema over his lower extremities but was without dyspnea. He had no fever or symptoms indicating an infection. No neurological examination was performed. His blood pressure was measured several times during the hospitalization, but was 123/89 mm Hg shortly after admission, with a pulse rate of 113 and a saturation of 93% without oxygen supply. His hemoglobin count was 170 gram/L (reference, 134–170), his erythrocyte count was 5.5 (× 10^12^/L) (reference, 4.2-5.7), his leukocyte count was 8.0 (× 10^9^/L) (reference, 3.5-8.8), and his thrombocyte count was 117 (× 10^9^/L) (reference, 145–348). Liver parameters were mostly normal (aspartate aminotransferase, alanine aminotransferase, gamma-glutamyl transferase, and bilirubin), except for hypoalbuminemia (24 g/L, reference, 34–45). His renal function was not impaired, as made evident by a normal plasma creatinine and a calculated glomerular filtration rate (GFR) of 75 mL/minute (reference, > 60 mL/minute). Following the first round of chemotherapy, he developed partly therapy-resistant back pain, and was planned for external radiation directed at his pelvis and lower back for palliative purposes. Besides the chemotherapy treatment, he was administered fentanyl (75 micrograms/hour, transdermal administration) for his back pain, ondansetron (4–8 mg intravenously) against chemotherapy-induced nausea, and sodium phosphate laxatives against morphine-instigated constipation. He was also administered furosemide intravenously (20–40 mg) when needed. Cortisone (betamethasone) was also administered orally using a standardized gradual reduction scheme.

**Fig. 2:**
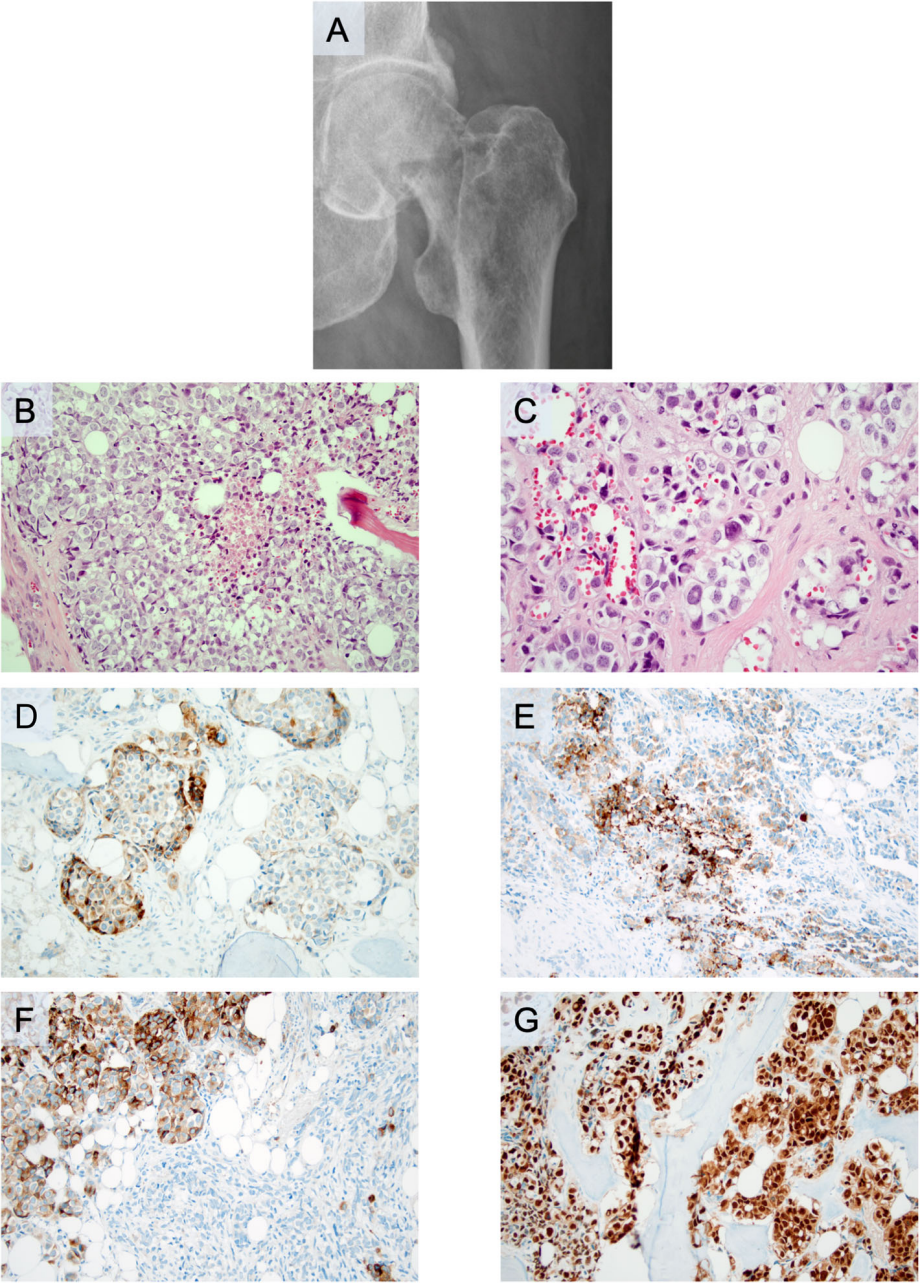
Radiological and microscopic findings of the subsequent surgical excision from a pathological hip fracture caused by a metastatic malignant melanoma with neuroendocrine differentiation. All microscopic images are magnified × 200 unless otherwise stated. **a**. Representative plain radiology scan displaying the pathological hip fracture from which the metastatic melanoma was diagnosed. **b**. Routine hematoxylin and eosin staining. Note the comedo-like necrosis in the central area. **c**. Routine hematoxylin and eosin at × 400 magnification, illustrating the nest-forming tumor with nuclear inclusions. **d**. Focal synaptophysin immunoreactivity (subsets of tumor cells). **e**. Focal melanoma antigen immunoreactivity (subsets of tumor cells). **f**. Focal human melanoma black 45 immunoreactivity (subsets of tumor cells). **g**. Diffuse nuclear SOX10 expression

Shortly afterwards, he developed a left-sided, pathological hip fracture (Fig. 2a). Following surgery, the excised femoral head and neck was sent for histopathological examination. Histological evaluation of material decalcified by formic acid and microwave treatment revealed infiltration by a nest-forming tumor with large atypical nuclei displaying multiple nucleoli and a partly loose chromatin (Fig. 2b, c). Nuclear inclusions were noted. The mitotic rate was 20 mitoses/10 high-power fields. Tumor necrosis was noted (Fig. 2b). Immunohistochemistry revealed partial expression of SYP, Melan A, and HMB45 (Table 1; Fig. 2d–f), as well as positive immunoreactivity towards SOX10 (Fig. 2g). Moreover, vimentin immunoreactivity was noted, as well as absent staining of CK MNF 116, CK7, CK20, desmin, myogenin, CD10, and CgA (data not shown). The diagnosis was consistent with a metastatic epithelioid malignant melanoma with neuroendocrine differentiation.

He subsequently developed bilateral pleural effusions, and despite intermittent treatment with pleurocentesis, he developed respiratory failure, which was aggravated by the occurrence of lobar pneumonia. Given his status, no clinical investigations regarding the occurrence of an undiagnosed primary cutaneous or mucosal malignant melanoma were initiated. Ultimately, he developed septicemia and died only 3 months after initial presentation. No autopsy was performed.

## Discussion and conclusions

We present a patient initially diagnosed as having metastatic NEC of unknown primary as dictated by the neuroendocrine markers of both first generation (SYP/CD56) and second generation (INSM1 and ISLET1) observed at histopathological investigations from a tumor biopsy. Intriguingly, standard melanoma markers (Melan A, HMB45) were negative at initial presentation, but subsequently positive in following investigations of a separate metastatic lesion in which more tissue was available for immunohistochemical assessment. The latter investigation rendered a diagnosis of malignant melanoma with neuroendocrine differentiation, which is a rare manifestation in the clinical setting. The case exemplifies how immunohistochemical patterns and routine morphology can differ within metastatic deposits and serves as a lesson to cautiously interpret neuroendocrine markers in the clinical context.

Malignant melanoma with neuroendocrine differentiation is rarely encountered, and the bulk of scientific data available is derived from case reports and small case series [6, 10–15]. In the largest study to date, Romano and co-workers described SYP positivity in 10 out of 34 malignant melanomas, typically only in small subsets of tumor cells [14]. Interestingly, neuroendocrine differentiation was particularly evident in epithelioid malignant melanomas, similar to our case. Other reports describe scattered immunoreactivity for SYP and CD56, but rarely CgA [10–13, 15]. In our case, the tumor also exhibited widespread CD56 immunoreactivity, focal nuclear ISLET1 staining adjoined by a puzzling cytoplasmic INSM1 pattern – all neuroendocrine markers, of which the latter two exhibit high specificity in previous publications [16, 17]. A possible differential diagnosis besides metastatic NEC includes metastatic Merkel cell carcinoma (MCC); however, the tumor was negative for both CK20 and CgA, and the Ki-67-index was much lower than usually observed in MCCs. Moreover, the combination of positivity for Melan A, HMB45, and SOX10 strongly argued in favor of malignant melanoma as opposed to metastatic tumors of other origin [18–21].

From a clinical perspective, neuroendocrine differentiation in disseminated malignant melanomas might confuse the responsible pathologist into rendering a diagnosis of metastatic NEC, as in our case. The overall histomorphology is suboptimal in core-needle biopsy material compared to routine sections of surgically excised material, which is clearly demonstrated in our case (Fig. 1a, b and Fig. 2b, c). In the excised material, the level of nuclear details is in fact enough to suspect a malignant melanoma – which is hardly the case by judging the hematoxylin and eosin (H&E) sections from the core-needle biopsy. Moreover, the immunohistochemical analyses varied to some extent when comparing the core-needle biopsy with the excised material, which could be due to differences in the sheer tumor area covered. Intriguingly, the heterogeneity of the tumor staining is perhaps best exemplified by the negative Melan A and HMB45 immunostaining results when assessing the chest wall core-needle biopsy (Fig. 1g, h), as compared to the excised femoral head and neck displaying focal but distinct immunoreactivity for both markers (Fig. 2e, f). However, given the widespread SOX10 immunoreactivity in the excised material (Fig. 2g), a lesson for future cases might be to always include SOX10 immunohistochemistry when assessing metastatic tumors with equivocal immunohistochemical findings regarding neuroendocrine markers.

Given the shared embryologic origin and functional aspects of melanocytes, neurons, and neuroendocrine cells, the notion that these cell types might share expressional profiles regarding vesicle transportation (SYP) and cell-cell adhesion (CD56) is not entirely inappropriate. However, given the general lack of published literature regarding neuroendocrine differentiation in malignant melanoma, little is known regarding patient outcome compared to conventional malignant melanomas. Although the coupling to prognosis is unclear, a general awareness among histopathologists regarding the potential for non-NETs to present with neuroendocrine features is warranted in order to secure the correct diagnosis. Treatment-wise, if a diagnosis of malignant melanoma would have been put forward earlier, the administration of carboplatin and etoposide would, in our case, probably have been avoided in favor for standardized melanoma protocols, irrespectively of neuroendocrine differentiation or not.

We conclude that surgical pathologists should interpret neuroendocrine markers with caution, as numerous non-NET variants have been reported in the literature, thereby potentially acting as clinical mimickers – which might lead to suboptimal treatment regimes. This is especially true from biopsy material, which is often scarce and thereby limits the number of immunohistochemical stains that can be ordered.

## Data Availability

All data generated or analyzed during this study are included in this published article.

## Abbreviations

CDX2: Caudal type homeobox 2
CEA: Carcinoembryonic antigen
CgA: Chromogranin A
CK MNF 116: Pan-cytokeratin
CT: Computed tomography
CUP: Cancer of unknown primary
EMA: Epithelial membrane antigen
GATA3: GATA binding protein 3
GLP1: Glucagon-like peptide 1
GFR: Glomerular filtration rate
H&E: Hematoxylin and eosin
HMB45: Human melanoma black 45
INSM1: Insulinoma-associated protein 1
ISLET1: ISL LIM homeobox 1
MCC: Merkel cell carcinoma
Melan A: Melanoma antigen
NCAM: Neural cell adhesion molecule
NEC: Neuroendocrine carcinoma
NET: Neuroendocrine tumor
PAX8: Paired box 8
PDX1: Pancreatic and duodenal homeobox 1
PSA: Prostate-specific antigen
SF1: Steroidogenic factor 1
SYP: Synaptophysin
TTF1: Thyroid transcription factor 1

## References

[1] Weltgesundheitsorganisation, Lloyd RV, Osamura RY, Klöppel G, Rosai J, International Agency for Research on Cancer. WHO classification of tumours of endocrine organs. 4th ed. Lyon: International Agency for Research on Cancer; 2017. p. 355. (World Health Organization classification of tumours).

[2] Kardoust Parizi M, Iwata T, Kimura S, Janisch F, Abufaraj M, Karakiewicz PI, *et al.* Focal neuroendocrine differentiation of conventional prostate adenocarcinoma as a prognostic factor after radical prostatectomy: a systematic review and meta-analysis. Int J Mol Sci. 2019;20(6):1374. 10.3390/ijms20061374 PMC6471399 30893781

[3] Bogina G, Munari E, Brunelli M, Bortesi L, Marconi M, Sommaggio M, et al. Neuroendocrine differentiation in breast carcinoma: clinicopathological features and outcome. Histopathology. 2016;68(3):422–32. 10.1111/his.12766 26114478

[4] Weissferdt A, Moran CA. Neuroendocrine differentiation in thymic carcinomas: a diagnostic pitfall: an immunohistochemical analysis of 27 cases. Am J Clin Pathol. 2016;145(3):393–400. 10.1093/ajcp/aqv095 27124922

[5] Yu L, Lao IW, Wang J. Epithelioid rhabdomyosarcoma: a clinicopathological study of seven additional cases supporting a distinctive variant with aggressive biological behaviour. Pathology. 2015;47(7):667–72. 10.1097/PAT.0000000000000321 26517641

[6] Banerjee SS, Eyden B. Divergent differentiation in malignant melanomas: a review. Histopathology. 2008;52(2):119–29. 10.1111/j.1365-2559.2007.02823.x 17825057

[7] O’Connor DT, Frigon RP, Sokoloff RL. Human chromogranin A. Purification and characterization from catecholamine storage vesicles of human pheochromocytoma. Hypertension. 1984;6(1):2–12. 10.1161/01.hyp.6.1.2 6693145

[8] Wiedenmann B, Franke WW. Identification and localization of synaptophysin, an integral membrane glycoprotein of Mr 38,000 characteristic of presynaptic vesicles. Cell. 1985;41(3):1017–28. 10.1016/s0092-8674(85)80082-9 3924408

[9] Bixby JL, Pratt RS, Lilien J, Reichardt LF. Neurite outgrowth on muscle cell surfaces involves extracellular matrix receptors as well as Ca2+-dependent and -independent cell adhesion molecules. Proc Natl Acad Sci U S A. 1987;84(8):2555–9. 10.1073/pnas.84.8.2555 PMC304693 3031688

[10] Banerjee SS, Menasce LP, Eyden BP, Brain AN. Malignant melanoma showing ganglioneuroblastic differentiation: report of a unique case. Am J Surg Pathol. 1999;23(5):582–8. 10.1097/00000478-199905000-00013 10328091

[11] Coli A, Giacomini PG, Bigotti G, Ferraro S, Alessandrini M, Del Vecchio M, *et al.* Aberrant neurofilament protein and synaptophysin expression in malignant melanoma of the nasal cavity. Histopathology. 2004;44(2):193–5. 10.1111/j.1365-2559.2004.01784.x 14764067

[12] Eyden B, Pandit D, Banerjee SS. Malignant melanoma with neuroendocrine differentiation: clinical, histological, immunohistochemical and ultrastructural features of three cases. Histopathology. 2005;47(4):402–9. 10.1111/j.1365-2559.2005.02240.x 16178895

[13] Ilardi G, Caroppo D, Varricchio S, Vita G, Di Lorenzo P, Insabato L, *et al.* Anal melanoma with neuroendocrine differentiation: report of a case. Int J Surg Pathol. 2015;23(4):329–32. 10.1177/1066896915573568 25722317

[14] Romano RC, Carter JM, Folpe AL. Aberrant intermediate filament and synaptophysin expression is a frequent event in malignant melanoma: an immunohistochemical study of 73 cases. Mod Pathol. 2015;28(8):1033–42. 10.1038/modpathol.2015.62 26022451

[15] Katerji H, Childs JM, Bratton LE, Peyre CG, Huber AR. Primary esophageal melanoma with aberrant CD56 expression: a potential diagnostic pitfall. Case Rep Pathol. 2017;2017:9052637. 10.1155/2017/9052637 PMC5694584 29230340

[16] Schmitt AM, Riniker F, Anlauf M, Schmid S, Soltermann A, Moch H, *et al.* Islet 1 (Isl1) expression is a reliable marker for pancreatic endocrine tumors and their metastases. Am J Surg Pathol. 2008;32(3):420–5. 10.1097/PAS.0b013e318158a397 18300808

[17] Rosenbaum JN, Guo Z, Baus RM, Werner H, Rehrauer WM, Lloyd RV. INSM1: a novel immunohistochemical and molecular marker for neuroendocrine and neuroepithelial neoplasms. Am J Clin Pathol. 2015;144(4):579–91. 10.1309/AJCPGZWXXBSNL4VD 26386079

[18] Ordóñez NG. Value of melanocytic-associated immunohistochemical markers in the diagnosis of malignant melanoma: a review and update. Hum Pathol. 2014;45(2):191–205. 10.1016/j.humpath.2013.02.007 23648379

[19] Shin J, Vincent JG, Cuda JD, Xu H, Kang S, Kim J, et al. Sox10 is expressed in primary melanocytic neoplasms of various histologies but not in fibrohistiocytic proliferations and histiocytoses. J Am Acad Dermatol. 2012;67(4):717–26. 10.1016/j.jaad.2011.12.035 22325460

[20] Cimino-Mathews A, Subhawong AP, Elwood H, Warzecha HN, Sharma R, Park BH, *et al.* Neural crest transcription factor Sox10 is preferentially expressed in triple-negative and metaplastic breast carcinomas. Hum Pathol. 2013;44(6):959–65. 10.1016/j.humpath.2012.09.005 PMC3978178 23260325

[21] Cancer Of unknown primary. [Internet]. University of Texas MD Anderson Cancer Center; 2018. Available from: https://www.mdanderson.org/content/dam/mdanderson/documents/for-physicians/algorithms/cancer-treatment/ca-treatment-unknown-primary-web-algorithm.pdf.

